# Mother−child histocompatibility and risk of rheumatoid arthritis and systemic lupus erythematosus among mothers

**DOI:** 10.1038/s41435-018-0055-7

**Published:** 2019-01-12

**Authors:** Giovanna I. Cruz, Xiaorong Shao, Hong Quach, Diana Quach, Kimberly A. Ho, Kirsten Sterba, Janelle A. Noble, Nikolaos A. Patsopoulos, Michael P. Busch, Darrell J. Triulzi, Nektarios Ladas, Rainer Blasczyk, Wendy S. W. Wong, Benjamin D. Solomon, John E. Niederhuber, Lindsey A. Criswell, Lisa F. Barcellos

**Affiliations:** 1 Genetic Epidemiology and Genomics Lab, Division of Epidemiology, School of Public Health, University of California Berkeley, 324 Stanley Hall, Berkeley, CA 94720-3220 USA; 2 Rosalind Russell/Ephraim P. Engleman Rheumatology Research Center, Department of Medicine, University of California San Francisco, San Francisco, CA 94143 USA; 3 Children’s Hospital Oakland Research Institute, 5700 M.L.K. Jr. Way, Oakland, CA 94609 USA; 4 Division of Genetics, Department of Medicine, Brigham & Women’s Hospital, Harvard Medical School, 77 Avenue Louis Pasteur, Boston, MA 02115 USA; 5 Program in Translational Neuropsychiatric Genomics, Institute for the Neurosciences, Department of Neurology, Brigham & Women’s Hospital, 75 Francis Street, Boston, MA 02115 USA; 6 Program in Medical and Population Genetics, Broad Institute of Massachusetts Institute of Technology and Harvard, 415 Main Street, Cambridge, MA 02142 USA; 7 Blood Systems Research Institute, 270 Masonic Avenue, San Francisco, CA 94118-4417 USA; 8 Institute for Transfusion Medicine, Department of Pathology, University of Pittsburgh, 3636 Blvd. of the Allies, Pittsburgh, PA 15213 USA; 9 Institute for Transfusion Medicine, Hannover Medical School, Hannover, Germany; 10 Division of Medical Genomics, Inova Translational Medicine Institute, 8110 Gatehouse Road, Falls Church, VA 22042 USA; 11 School of Medicine, Johns Hopkins University, Baltimore, MD 21205 USA; 12 California Institute for Quantitative Biosciences (QB3), University of California Berkeley, 174 Stanley Hall, Berkeley, CA 94720-3220 USA

**Keywords:** Disease genetics, Immunogenetics

## Abstract

The study objective was to test the hypothesis that having histocompatible children increases the risk of rheumatoid arthritis (RA) and systemic lupus erythematosus (SLE), possibly by contributing to the persistence of fetal cells acquired during pregnancy. We conducted a case control study using data from the UC San Francisco Mother Child Immunogenetic Study and studies at the Inova Translational Medicine Institute. We imputed human leukocyte antigen (HLA) alleles and minor histocompatibility antigens (mHags). We created a variable of exposure to histocompatible children. We estimated an average sequence similarity matching (SSM) score for each mother based on discordant mother−child alleles as a measure of histocompatibility. We used logistic regression models to estimate odds ratios (ORs) and 95% confidence intervals. A total of 138 RA, 117 SLE, and 913 control mothers were analyzed. Increased risk of RA was associated with having any child compatible at *HLA-B* (OR 1.9; 1.2–3.1), *DPB1* (OR 1.8; 1.2–2.6) or *DQB1* (OR 1.8; 1.2–2.7). Compatibility at mHag ZAPHIR was associated with reduced risk of SLE among mothers carrying the HLA-restriction allele B*07:02 (*n* = 262; OR 0.4; 0.2–0.8). Our findings support the hypothesis that mother−child histocompatibility is associated with risk of RA and SLE.

## Introduction

Rheumatoid arthritis (RA) and systemic lupus erythematosus (SLE) are two prototypic autoimmune conditions. Despite differing clinical manifestations, a common feature is the production of B- and T-cell responses directed against self-antigens. Both diseases have strong associations with human leukocyte antigen (HLA) alleles [1] and predominately affect women [2, 3]. In RA, the female to male ratio is 3:1 and 9:1 in SLE [3]. The sex disparity has prompted investigation into various female-specific exposures, including those related to pregnancy [2], as potential etiologic factors.

The effect of pregnancy on RA and SLE activity is well documented and supports the hypothesis that immunologic changes during pregnancy are implicated in RA and SLE disease processes. The improvement of RA symptoms with pregnancy has been noted for decades [4, 5]. In SLE, relative to nonpregnant patients, pregnant patients have higher disease activity scores [6] and experience more flares [7]. Risk of RA increases during the first 2 years after birth [8–10]. In contrast to RA, a recent birth is associated with a reduction in risk of developing SLE lasting up to 10 years [11]. HLA disparity has been associated with improvement of RA symptoms during the course of pregnancy [12–14] and with delayed onset of RA post-pregnancy [13]. Persistent fetal microchimerism is more common in RA and SLE patients compared to controls [15]. One hypothesis is that the persistence of fetal cells in the mother’s body may be analogous to a graft and its immunological effect may be mediated by HLA relationships between mother and fetus [16].

The effects of HLA mismatch have been extensively investigated in the context of transplantation. HLA mismatch is associated with increased risk of graft-versus-host disease (GVHD) [17] as is mismatching on minor histocompatibility antigens (mHags) among HLA-matched donor−recipients [18]. mHags are polymorphic peptides encoded by genes outside of the HLA region that are presented in an HLA-restricted manner, meaning that the peptide binds to specific HLA molecules [19]. The similarities between GVHD and the clinical manifestations of certain autoimmune conditions have led to the hypothesis that mother−child HLA mismatch may contribute to female autoimmune disease processes [20, 21]. However, these studies have been limited to the investigation of a small number of HLA loci. The objective of this study was to investigate the association between RA and SLE and mother−child histocompatibility at seven classical HLA loci and six HLA-restricted mHags in a cohort of mother−child pairs.

## Results

RA and SLE cases were older at the time of interview or electronic medical record (EMR) extraction compared to controls (Table 1). On average, RA cases were diagnosed at age 39 and SLE cases at age 42. Cases had a greater number of live births compared to the average number among controls.

**Table 1:** Characteristics of mothers by rheumatoid arthritis (RA), systemic lupus erythematosus (SLE) and control status

Characteristics	RA	SLE	Controls
*N*^a^	138	117	913
Age at interview, mean ± SD	56.0 ± 9.2	56.3 ± 10.7	36.5 ± 9.2
Number of live births, mean ± SD	2.2 ± 1.0	2.4 ± 1.2	1.9 ± 0.9
Number of live births prior to diagnosis, mean ± SD	2.0 ± 1.0	2.2 ± 1.2	—
Years between last birth before diagnosis and diagnosis, mean ± SD^b^	13.5 ± 11.0	16.1 ± 10.4	—
Age at diagnosis, mean ± SD	39.4 ± 11.6	42.7 ± 10.6	—

^a^Participants included in HLA-compatibility analyses

^b^Age at last birth before diagnosis available for 120 RA cases

### Mother−child histocompatibility

Over the seven individual HLA loci tested in RA and SLE separately, *HLA-B*, *DPB1* and *DQB1* were associated with increased risk of RA when mothers had one or more children prior to diagnosis who were histocompatible from the mother’s perspective (Table 2). The association between *HLA-B* and RA was odds ratio (OR) 1.9; 95% confidence interval (CI), 1.2–3.1, between *DPB1* and RA OR 1.8; 95% CI, 1.2–2.6 and OR 1.8; 95% CI, 1.2–2.7 between *DQB1* and RA. Among SLE cases, there was no evidence of an association between having any histocompatible children at any of the classical HLA loci tested and risk of disease (Table 3). A minority of cases and controls had children who were histocompatible at all seven loci (3.6% of RA cases, 1.7% of SLE, and 0.8% of controls). We found a significant difference between cases and controls and the proportion of missing histocompatibility status at *DQB1* (controls 6%, RA 16%, SLE 25% missing) and between SLE status and *HLA-A* (controls 3% and SLE 6%). No other differences were found at the other loci tested.

**Table 2:** Association between one or more children histocompatible from the mother’s perspective among rheumatoid arthritis (RA) cases compared to controls (analyses are for each HLA locus and are adjusted for number of live births)

Any histocompatible children	RA *n* (%)	Controls *n* (%)	OR (95% CI)	Bonferroni corrected *p* value^a^
HLA Class I				
A				
None	104 (75)	683 (75)	Reference	
1 or more	34 (25)	230 (25)	1.0 (0.6–1.5)	1.00
B				
None	111 (80)	814 (89)	Reference	
1 or more	27 (20)	99 (11)	1.9 (1.2–3.1)	0.04
C				
None	105 (76)	752 (82)	Reference	
1 or more	33 (24)	161 (18)	1.4 (0.9–2.2)	0.77
HLA Class II				
DPB1				
None	63 (46)	555 (61)	Reference	
1 or more	75 (54)	358 (39)	1.8 (1.2–2.6)	0.01
DQA1				
None	74 (54)	597 (65)	Reference	
1 or more	64 (46)	316 (35)	1.6 (1.1–2.3)	0.10
DQB1				
None	93 (67)	725 (79)	Reference	
1 or more	45 (33)	188 (21)	1.8 (1.2–2.7)	0.03
DRB1				
None	103 (75)	765 (84)	Reference	
1 or more	35 (25)	148 (16)	1.6 (1.0–2.4)	0.28

*OR* odds ratio, *95% CI*, confidence interval

^a^Bonferroni corrected *p* values for seven tests

**Table 3:** Association between one or more children histocompatible from the mother’s perspective among systemic lupus erythematosus (SLE) cases compared to controls (analyses are for each HLA locus and are adjusted for number of live births)

Any histocompatible children	SLE *n* (%)	Controls *n* (%)	OR (95% CI)	Bonferroni corrected *p* value^a^
HLA Class I				
A				
None	78 (67)	683 (75)	Reference	
1 or more	39 (33)	230 (25)	1.5 (1.0–2.3)	0.35
B				
None	101 (86)	814 (89)	Reference	
1 or more	16 (14)	99 (11)	1.2 (0.7–2.2)	1.00
C				
None	92 (79)	752 (82)	Reference	
1 or more	25 (21)	161 (18)	1.2 (0.7–1.9)	1.00
HLA Class II				
DPB1				
None	64 (55)	555 (61)	Reference	
1 or more	53 (45)	358 (39)	1.3 (0.9–1.9)	1.00
DQA1				
None	69 (59)	597 (65)	Reference	
1 or more	48 (41)	316 (35)	1.2 (0.8–1.9)	1.00
DQB1				
None	85 (73)	725 (79)	Reference	
1 or more	32 (27)	188 (21)	1.4 (0.9–2.1)	1.00
DRB1				
None	103 (75)	765 (84)	Reference	
1 or more	35 (25)	148 (16)	1.3 (0.8–2.2)	1.00

*OR* odds ratio, *95% CI* confidence interval

^a^Bonferroni corrected *p* values for seven tests

Results using the SSM score to categorize mother−child compatibility at each locus are found in Tables 4 and 5. The SSM score range varied across HLA loci. Supplementary Figures 1 to 7 present the SSM score distribution at each locus by disease status. *DQA1* had the smallest range in score and *HLA-A* and *B* had the greatest. Results for cases and controls were very similar (Supplementary Table 4). All estimates from the multivariable models comparing the top quartile of similarity to the least and their association with RA and SLE contained the null (Tables 4 and 5). The SSM score was correlated with our allele-based definition of histocompatibility where average scores were significantly lower in the histocompatible group, as expected (data not shown).

**Table 4:** Mean sequence similarity matching (SSM) score and risk of rheumatoid arthritis (RA) among mothers

Histocompatibility per SSM score (Quartile)	RA *n* (%)	Controls *n* (%)	OR (95% CI)^a^	*p* for linear trend
HLA Class I				
A				
Least (4th)	30 (22)	222 (24)	Reference	
Most (1st)	35 (25)	229 (25)	1.2 (0.7–2.0)	0.35
B				
Least (4th)	37 (27)	231 (25)	Reference	
Most (1st)	34 (25)	231 (25)	1.0 (0.6–1.6)	0.99
C				
Least (4th)	35 (25)	209 (23)	Reference	
Most (1st)	32 (23)	227 (25)	0.8 (0.5–1.4)	0.52
HLA Class II				
DPB1				
Least (4th)	24 (17)	220 (24)	Reference	
Most (1st)	42 (30)	244 (27)	1.5 (0.9–2.6)	0.26
DQA1				
Least (4th)	22 (16)	218 (24)	Reference	
Most (1st)	24 (17)	221 (24)	1.1 (0.6–2.0)	0.65
DQB1				
Least (4th)	28 (20)	295 (32)	Reference	
Most (1st)	33 (24)	221 (24)	1.6 (0.9–2.8)	0.04
DRB1				
Least (4th)	23 (17)	210 (23)	Reference	
Most (1st)	34 (25)	244 (27)	1.3 (0.7–2.3)	0.65

*OR* odds ratio, *95% CI* confidence interval

^a^Adjusted for number of live births. The SSM score estimates degree of similarity between two alleles. A lower score indicates increased similarity relative to a higher score; therefore, the fourth quartile of scores is intended to represent the least histocompatible group and the first quartile the most

**Table 5:** Mother−child similarity score—Mean sequence similarity matching (SSM) score and risk of systemic lupus erythematosus (SLE) among mothers

Histocompatibility per SSM score (Quartile)	SLE *n* (%)	Controls *n* (%)	OR (95% CI)^a^	*p* for linear trend
HLA Class I				
A				
Least (4th)	26 (22)	222 (24)	Reference	
Most (1st)	31 (27)	229 (25)	1.2 (0.7–2.2)	0.46
B				
Least (4th)	23 (20)	231 (25)	Reference	
Most (1st)	37 (32)	231 (25)	1.5 (0.9–2.7)	0.23
C				
Least (4th)	30 (26)	209 (23)	Reference	
Most (1st)	26 (22)	227 (25)	0.8 (0.4–1.4)	0.19
HLA Class II				
DPB1				
Least (4th)	26 (22)	220 (24)	Reference	
Most (1st)	19 (16)	244 (27)	0.7 (0.3–1.2)	0.04
DQA1				
Least (4th)	26 (22)	218 (24)	Reference	
Most (1st)	19 (16)	221 (24)	0.7 (0.4–1.4)	0.25
DQB1				
Least (4th)	29 (25)	295 (32)	Reference	
Most (1st)	18 (15)	221 (24)	0.8 (0.4–1.5)	0.89
DRB1				
Least (4th)	14 (12)	210 (23)	Reference	
Most (1st)	28 (24)	244 (27)	1.7 (0.9–3.3)	0.42

*OR* odds ratio, *95% CI* confidence interval

^a^Adjusted for number of live births. The SSM score estimates degree of similarity between two alleles. A lower score indicates increased similarity relative to a higher score; therefore, the fourth quartile of scores is intended to represent the least histocompatible group and the first quartile the most

### Minor histocompatibility antigen compatibility

Table 6 shows results for association tests between mHag compatibility versus incompatibility and SLE among mothers. The number of mothers who carried the HLA restriction allele for each mHag determined the sample size for each model (Table 6). We found evidence of reduced SLE risk associated with having one or more children who were compatible for ZAPHIR, restricted for HLA-B*07:02, regardless of mother−child histocompatibility status; OR 0.4; 95% CI, 0.2–0.8. Taking HLA compatibility into account (Table 7), SLE cases were less likely to have HLA-compatible/mHag compatible children (yes/no) at ZAPHIR compared to controls (0% vs. 9% respectively, Fisher’s exact *p* value = 0.05). However, HLA-compatibility combined with mHag compatibility at LB-WNK1 was more likely among cases than control mothers (23% vs. 13% respectively, Fisher’s exact *p* value = 0.02). We did not observe associations between compatibility and SLE at other mHags investigated.

**Table 6:** Mother−child compatibility at minor histocompatibility antigens (mHag) and risk of systemic lupus erythematosus (SLE) among mothers with the corresponding HLA restriction

Any mHag-matched children	HLA restriction	SLE *n* (%)	Controls *n* (%)	OR (95% CI)^a^	*p*
SLC19A1	DRB1*15:01				
None		24 (52)	111 (53)	Reference	
1 or more		22 (48)	98 (47)	0.9 (0.5–1.7)	0.76
LB-WNK1	A*02:01				
None		45 (47)	252 (57)	Reference	
1 or more		50 (53)	188 (43)	1.5 (0.9–2.3)	0.11
HA-3	A*01:01				
None		32 (48)	152 (52)	Reference	
1 or more		34 (52)	140 (48)	1.2 (0.7–2.1)	0.50
ZAPHIR	B*07:02				
None		29 (74)	117 (52)	Reference	
1 or more		10 (26)	106 (48)	0.4 (0.2–0.8)	0.01
HEATR1	B*08:01				
None		31 (54)	127 (64)	Reference	
1 or more		26 (46)	73 (37)	1.5 (0.8–2.7)	0.22
C19orf48	A*02:01				
None		44 (46)	245 (56)	Reference	
1 or more		51 (54)	195 (44)	1.4 (0.9–2.3)	0.13

*OR* odds ratio, *95% CI* confidence interval

^a^Adjusted for number of live births

**Table 7:** Fisher’s Exact test between having any children who are mHag and HLA compatible and risk of systemic lupus erythematosus (SLE) among mothers (histocompatible from the mother’s perspective at the restriction HLA locus and at the minor histocompatibility antigen)

Any HLA-matched/mHag-matched children	HLA restriction	SLE *n* (%)	Controls *n* (%)	*p*^a^
SLC19A1	DRB1*15:01			
None		44 (96)	189 (90)	
1 or more		2 (4)	20 (10)	0.39
LB-WNK1	A*02:01			
None		73 (77)	384 (87)	
1 or more		22 (23)	56 (13)	0.02
HA-3	A*01:01			
None		57 (86)	251 (86)	
1 or more		9 (14)	41 (14)	1.0
ZAPHIR	B*07:02			
None		39 (100)	202 (91)	
1 or more		0 (0)	21 (9)	0.05
HEATR1	B*08:01			
None		51 (89)	189 (95)	
1 or more		6 (11)	11 (5)	0.22
C19orf48	A*02:01			
None		75 (79)	366 (83)	
1 or more		20 (21)	74 (17)	0.37

^a^Fisher’s exact test *p* value

## Discussion

Our study addressed the hypothesis that mother−child histocompatibility increases risk of RA and SLE possibly by facilitating the persistence of fetal cells. We found that RA cases were more likely to have a child histocompatible at *HLA-B*, *DPB1* and *DQB1* compared to controls. Our findings are consistent with previous studies that found an association between decreased disease activity during pregnancy and *DQA1* and *DQB1* incompatibility [12, 13]. One hypothesis is that incompatibility results in competition between fetal and maternal self-antigens, where one displaces the other leading to an improvement of symptoms or delayed onset. Compatibility may increase risk of disease by contributing to the process of epitope spreading prior to disease onset [22], if the quantity of risk-associated antigens increases with pregnancy. Compatibility may diminish T-reg activation that helps to shift the balance of Th1/Th2 cytokines. Paternal antigens induce T-regs independent of pregnancy hormones in murine models of pregnancy [23].

We found no association between histocompatibility and SLE. The lack of consistency between the two diseases does not support the hypothesis that histocompatibility functions as a common mechanism in autoimmunity, such as by facilitating the persistence of fetal cells. Our findings do not rule out that histocompatibility contributes to autoimmunity through other mechanisms and highlight the importance of considering the contrasting pattern of the effect of pregnancy on RA and SLE [24]. The findings that incompatibility is associated with remission and delayed onset are consistent with a mechanism of action based on increased production of T-regs during pregnancy that shifts the Th1/Th2 balance in RA. Lupus flares during pregnancy are more likely in women with active disease at the time of pregnancy [25]. In lupus pregnancy, complement activation has been implicated in flares during pregnancy [26], highlighting the distinct immunological pathways that lead to each disease.

HLA Class II incompatibility has been reported to be associated with remission or improvement of RA symptoms during pregnancy and possibly with risk of developing RA in the post-partum period [12, 13]. One study found increased risk of RA in the first year after birth was associated with incompatibility at *DQA1* (OR 3.86; 95% CI, 1.03–14.52) and *DQB1* (OR 4.23, 95% CI, 1.12–15.9) [13]. However, the study was based on a small sample of 16 women who developed RA during this period. Compatibility at HLA Class II has been associated with increased risk of scleroderma [27]. Among controls, the frequency of compatibility in our study at *DRB1* (17%) and *DQA1* (35%) was similar to the one reported in the scleroderma study (16 and 34% respectively) [27]. Estimates for cases differ according to disease, possibly due to relative differences in pathophysiology. Our findings are in agreement with a previous study that failed to find evidence of an association between mother−child histocompatibility at *DRB1* and SLE [28].

Compatibility at the minor histocompatibility antigen ZAPHIR was associated with decreased risk of SLE among mothers who carried the presenting HLA allele B*07:02 (OR 0.4; 95% CI, 0.2–0.8). Furthermore, we found a similar association when analyses were restricted to the group of mothers and children who were both histocompatible at *HLA-B* and ZAPHIR (*p* = 0.05). ZAPHIR was identified in the context of graft-versus-tumor effect in patients with renal cell carcinoma [29]. In the case of one patient who experienced a partial regression of lung metastasis after stem cell transplantation found that the presence of ZAPHIR-specific CD8+ T cells resulted in a graft-versus-tumor effect and prevented against GVHD. In SLE, these findings merit further investigation due to renal involvement in more severe disease.

SLE cases were more likely to be compatible at both LB-WNK1 and at its HLA-A restriction site compared to controls (*p* = 0.02). LB-WNK1 is encoded by WNK1 [19]. WNKs are kinases associated with hypertension and involved in the regulation of sodium and potassium ions and water status in the nephron [30]. Elevated risk of preeclampsia among SLE pregnancies compared to the general population, including prior to disease onset [31] supports a potential biological relevance for the observed association. WNK1 deficiency in mice increases conjugation between T cells and antigen-presenting cells and T cells home less efficiently to lymph nodes and spleen [32]. WNK1 has been found to be involved in the establishment of pregnancy and regulation of inflammatory cytokines [33]. Functional studies in SLE and pre-SLE pregnancies are needed to understand the significance of compatibility in the disease process.

Minor histocompatibility antigen disparity is immunogenic as evident from their involvement in GVHD among HLA-matched transplant recipients and in the potentially curative graft-versus-leukemia effect (GVL) [34]. The use of mismatched mHags as a way to trigger GVL is evidence that mHags can be manipulated for therapeutic applications [35]. As more mHags are discovered, their relevance and function is likely to depend on the context of the interaction.

This study is among the first investigations of mother−child mHag compatibility and risk of SLE. CD8+ T cells specific for minor histocompatibility antigens of fetal origin have been found in mothers up to 22 years after delivery [36] as have mHag-specific regulatory T cells (T-regs) [37]. Exposure to fetal mHags is likely to come from microchimerism or from particles that are shed from the placenta and enter the maternal circulation [38]. Minor histocompatibility antigens are expressed in placental material [39]. Placental vesicles of various sizes carry fetal nucleic acids, proteins and lipids and are hypothesized to have immunomodulatory effects [40, 41]. It is possible that antigens from fetal sources mediate the effect of pregnancy on RA and SLE. The shedding of placental material could overload the dysfunctional clearance mechanism in SLE patients, leading to exacerbation of symptoms [42]. Functional studies of maternal immune cell responses to fetal minor histocompatibility antigens are needed to understand their biological effect in the context of autoimmunity.

### Strengths and limitations

We describe a comprehensive report on mother−child histocompatibility and risk of RA and SLE. We conducted a case−control study in a well-characterized mother−child study of autoimmunity. The MCIS is a unique resource with genotype data for case and control families, detailed questionnaire and medical record abstracted clinical data. Our exposure definition of histocompatibility at both classical HLA loci and minor histocompatibility antigens was based on biology.

Some study limitations must be acknowledged. The use of SNP2HLA to impute alleles at two-field resolution for *HLA-A, B, C, DPB1, DQA1, DQB1* and *DRB1* resulted in different degrees of missing data by locus. This is possibly due to the use of data derived from different genotyping platforms. For the most part, missingness was not associated with case control status with the exception of *DQB1*. Both RA and SLE cases were more likely to have a missing value for histocompatibility at *DQB1* compared to controls. It is possible that the SNPs available for imputation of *DQB1* alleles for RA and SLE cases were not sufficient. For this reason, the association between histocompatibility at *DQB1* and RA and SLE requires further investigation. There were also more missing data for *HLA-A* histocompatibility and SLE but the difference between cases and controls was small (3%) and it is unlikely this difference would substantially impact results.

Inclusion of controls from pregnancy cohorts at ITMI resulted in a significant difference in the age at interview or EMR data extraction. It is possible that the younger age of controls could bias our results by including future cases. However, RA and SLE are both relatively rare in the general population and we expect similar rates apply to this cohort. Moreover, inclusion of future cases is likely to bias our results towards the null. Differences in data collection (self-administered questionnaire vs. EMR) could result in information bias. For our study, the only variable included in analyses was the number of live births. Studies have shown a high level of concordance in the number of live births reported by self-report and medical records (kappa = 1.0) [43, 44], therefore minimizing concern that the method of collection contributed to the results. The lower number of live births among controls compared to cases could reduce the probability of exposure. However, all models are adjusted for the number of live births.

In conclusion, we found new evidence for mother−child HLA-mediated effects in RA and SLE. A comprehensive evaluation of immunogenic peptides, such as minor histocompatibility antigens, is needed, as they may help explain pregnancy-related effects and may be promising therapeutic targets.

## Patients and methods

We conducted a case−control study of 3746 individuals including 1614 non-Hispanic white mothers and their 2132 children. All mothers had at least one child who provided blood or saliva for DNA extraction. Data for mothers and children were obtained from the University of California San Francisco (UCSF) Mother−Child Immunogenetic Study (MCIS) and research studies conducted at the Inova Translational Medicine Institute (ITMI), Inova Health System, Falls Church, Virginia. The MCIS enrolled RA and SLE case mothers who were identified from genetic studies of autoimmunity at UCSF. Only mothers with at least one child born prior to diagnosis were included in the study. Cases were ascertained by the most current American College of Rheumatology (ACR) criteria available at the time of enrollment. RA cases met the 1987 revised ACR criteria [45] and SLE cases met the 1997 revised ACR criteria [46, 47]. Control mothers with no prior history of RA or SLE and their children were identified from various sources. MCIS control mothers were recruited from blood donors who had participated in the Leukocyte Antibody Prevalence Study (LAPS) [48] at the Blood Centers of the Pacific and the Institute for Transfusion Medicine in Pittsburgh, PA. Data for mother−child control pairs were also obtained from the ITMI’s Molecular Study of Preterm Birth and the First 1000 Days of Life studies. Family trios composed of a neonate and both parents were recruited at the Inova Women’s Hospital, Inova Fairfax Medical Center, Falls Church, Virginia [49]. We included mother−child pairs from ITMI cohorts if the mother was of European ancestry and had no prior history of RA or SLE. All participants provided written informed consent. The study protocol is in accordance with the Declaration of Helsinki and was approved by the UCSF and UC Berkeley Institutional Review Boards (IRB). The Western IRB and the Inova Health System IRB approved ITMI studies.

### Clinical and questionnaire data

For cases, we obtained the date of diagnosis and clinical characteristics from medical records. The MCIS collected data from case and control mothers on reproductive history and potential confounders through a self-administered questionnaire. For ITMI control mothers, reproductive history, mother’s and child’s date of birth and mother’s history of RA and SLE were obtained from electronic medical records (EMR).

### Genotyping

Cases were genotyped for a panel of 2360 SNPs in the MHC region using the combined MHC Exon-Centric and Mapping panels from Illumina. Full genome profiling of MCIS controls and children was conducted using the Illumina 660K SNP array or the Illumina ImmunoChip. QA/QC criteria were applied to all genotype data resulting in the exclusion of SNPs that were genotyped in <80% of samples and/or had a minor allele frequency (MAF) < 1%. All genotyping of MCIS participants was performed at UC Berkeley. Whole-genome sequencing (WGS) data were available for ITMI control mother−child duos. ITMI WGS and QA/QC methods have been previously described [49]. For the Molecular Study of Preterm Birth samples WGS, assembly and variant calling were performed by Complete Genomics (CG) (Mountain View, CA) using Complete Genomics’ Assembly Pipeline versions 2.0.0–2.03. The 1000 Days of Life Study samples were sequenced by Illumina (San Diego, CA) with the Illumina Whole Genome Sequencing Service Informatics Pipeline version 2.01–02. In addition, we excluded SNPs that were genotyped in <90% of samples and/or had an MAF < 1%. We verified familial relationships for all mother−child duos.

### HLA allele imputation

We used SNP2HLA [50] to impute HLA alleles using post-QA/QC genotype and WGS data. After imputation, we excluded variants with a low measure of imputation accuracy (*r*^2^ < 0.3). As an additional QC measure of imputation quality, we compared imputation results at the allelic level to typed two-field *DRB1* data available for a subset of MCIS participants (*n* = 2136) using the method described by Raychaudhuri et al. [51]. The imputation procedure correctly called 93.1% of alleles. Using a chi-square test and a significance threshold of *α* = 0.05, we compared allele accuracy by platform. We did not find statistically significant differences by platform for the *DRB1* alleles investigated (*p* = 0.19). The process of imputation and QA/QC steps resulted in different sample sizes available at each of the seven classical HLA loci (*HLA-A, B, C, DPB1, DQA1, DQB1*, and *DRB1*) at two-field resolution. We took a complete case approach in order to facilitate the interpretation of results.

### Minor histocompatibility antigens

We identified single nucleotide polymorphisms (SNPs) correlated with minor histocompatibility antigens (mHags) from a previously published report [52]. We obtained SNP data for MCIS SLE cases available from other genetic studies of autoimmunity at UCSF. ImmunoChip genotype data were available for SLE cases and their children for mHags SLC19A1 (rs1051266), LB-WNK1 (rs12828016), and ZAPHIR (rs2074071). In order to investigate as many mHags as possible, we imputed mHag SNPs using IMPUTE2 and the 1000 genomes (Phase 3) reference panel [53]. We excluded imputed SNPs with an info score < 0.3. After QA/QC, we had three additional SNPs for mothers and at least one child born prior to diagnosis: HA-3 (rs2061821), HEATR1 (rs2275687), C19orf48 (rs3745526). For an additional QA/QC measure of imputation accuracy, we compared the results of our imputed rs2061821 with genotyped data from Illumina’s 550K platform available for a subset of SLE cases and found 100% concordance. For ITMI controls, WGS data were available for all six SNPs. RA mothers were not included in this analysis due to the lack of datasets available to extract or impute the SNPs correlated with mHags.

### Classification of mother−child histocompatibility profiles

All children were classified into one of two histocompatibility categories at each classical HLA locus (*HLA-A, B, C, DPB1, DQA1, DQB1*, and *DRB1*) and SNPs for mHags SLC19A1, LB-WNK1, ZAPHIR, HA-3, HEATR1 and C19orf48. For case mothers, only children born before diagnosis were included. A child was classified as histocompatible from the mother’s perspective if his or her paternally inherited allele was indistinguishable from either of the mother’s alleles. Based on this classification, a binary variable for having any histocompatible children was generated for each mother at each HLA locus and mHag SNP.

### Histocompatibility defined by sequence similarity matching (SSM) score

In hematopoietic stem cell transplantation an exact match may be difficult to find. The risk of GVHD increases with the number of mismatched alleles [54]. In the case of a mismatch, it may be possible to reduce the risk of GVHD by estimating the allogeneic potential of the mismatch by selecting the least structurally different allele [55]. *HistoCheck* is a bioinformatics web tool that estimates an SSM based on amino acid sequence similarity [55]. This quantitative approach can be informative in estimating degree of similarity between the noninherited maternal allele and the paternal allele carried by the child. We estimated an SSM score for mother−child pairs who were not HLA-identical at each locus. Where mother and child did not differ at either allele, we assigned a score of zero. For each mother, we created an average SSM score at individual HLA loci. We categorized average SSM score into four groups according to values in the control group. Smaller values indicate increased similarity between mothers and children and are intended to represent greater degree of histocompatibility.

### Statistical analysis

Analyses for questions pertaining to mother−child histocompatibility at classical HLA loci were conducted in a sample of 138 RA, 117 SLE, and 913 control mothers and their respective children. We included mothers who had complete histocompatibility data at seven HLA loci (*HLA-A, B, C, DPB1, DQA1, DQB1, DRB1*). A total of 206 SLE cases and 965 controls and 1387 children were available for mHag-related analyses. Only cases with at least one child with data born prior to diagnosis were included. We used logistic regression models to estimate ORs and 95% CIs for the association between mother’s RA and SLE status and any histocompatibility at each HLA locus or mHag SNP and quartiles of average SSM score. We analyzed mHag compatibility in an HLA-restricted manner, meaning that only mothers who carried the HLA allele associated with the mHag were included. In addition, we tested whether case mothers were more likely to have any HLA-compatible/mHag compatible children compared to controls using Fisher’s exact test.

We used directed acyclic graphs to identify potential confounders and proceeded to include number of live births in our analyses. To correct for multiple testing, we applied a Bonferroni correction to classical HLA analyses to account for tests at seven loci [56]. To investigate the probability of introducing selection bias, we compared the proportion of missing data at each of the seven HLA loci by disease status using a chi-square test and considered a *p* value of <0.05 significant. We tested for the presence of a linear trend in our analyses of SSM score quartiles at each locus and autoimmune disease. Statistical analyses were conducted using Stata 13 (StataCorp, College Station, Texas) and R [57].

## Supplementary information

Supplementary Tables

Supplementary Figure 1. HLA-A Mother-Child average Sequence Similarity Matching (SSM) Score by disease status, (n=1,168)

Supplementary Figure 2. HLA-B Mother-Child average Sequence Similarity Matching (SSM) Score by disease status, (n=1,168)

Supplementary Figure 3. HLA-C Mother-Child average Sequence Similarity Matching (SSM) Score by disease status, (n=1,168)

Supplementary Figure 4. HLA-DPB1 Mother-Child average Sequence Similarity Matching (SSM) Score by disease status, (n=1,168)

Supplementary Figure 5. HLA-DQA1 Mother-Child average Sequence Similarity Matching (SSM) Score by disease status, (n=1,168)

Supplementary Figure 6. HLA-DQB1 Mother-Child average Sequence Similarity Matching (SSM) Score by disease status, (n=1,168)

Supplementary Figure 7. HLA-DRB1 Mother-Child average Sequence Similarity Matching (SSM) Score by disease status, (n=1,168)
