# Supplementary material for: Mother−child histocompatibility and risk of rheumatoid arthritis and systemic lupus erythematosus among mothers
Source: Genes Immun. 2019 Jan 12;21(1):27–36. doi: 10.1038/s41435-018-0055-7 (PMC7039805; doi:10.1038/s41435-018-0055-7)
Supplement: Supplementary file 1 — Supplementary Tables [file 41435_2018_55_MOESM1_ESM.docx]

**Supplementary Table 1.** Source of recruitment of mothers and children included in the histocompatibility analysis

| **Study source** | **RA** | **SLE** | **Control** |
| --- | --- | --- | --- |
| UCSF Mother-Child Immunogenetic Study | 138 | 117 | 132 |
| ITMI Molecular Study of Preterm Birth | -- | -- | 308 |
| ITMI First 1,000 Days of Life | -- | -- | 473 |

**Supplementary Table 2.** Accuracy of HLA genotype imputation by genotyping platform

| **Genotyping platform** | **Samples, n** | **Accuracy** |
| --- | --- | --- |
| Illumina MHC Exon-Centric and Mapping Panel | 374 | 95% |
| Illumina ImmunoChip | 1530 | 93% |
| Illumina 660K SNP Array | 220 | 95% |

**Supplementary Table 3.** Imputation accuracy for HLA-DRB1 alleles among MCIS Study, n=2,136

| **DRB1 alleles** | **Genotyped** | | **Imputed** | | **r** | **Accuracy** |
| --- | --- | --- | --- | --- | --- | --- |
|  | **Number** | **Frequency** | **Number** | **Frequency** |  |  |
| *01:01 | 399 | 9.3% | 400 | 9.4% | 0.97 | 99.5% |
| *01:02 | 39 | 0.9% | 42 | 1.0% | 0.97 | 99.9% |
| *01:03 | 52 | 1.2% | 47 | 1.1% | 0.87 | 99.7% |
| *03:01 | 573 | 13.4% | 574 | 13.4% | 1.00 | 100.0% |
| *04:01 | 445 | 10.4% | 536 | 12.5% | 0.89 | 97.6% |
| *04:02 | 39 | 0.9% | 39 | 0.9% | 1.00 | 100.0% |
| *04:03 | 21 | 0.5% | 0 | 0.0% | 0.00 | 99.5% |
| *04:04 | 189 | 4.4% | 192 | 4.5% | 0.95 | 99.5% |
| *04:05 | 59 | 1.4% | 57 | 1.3% | 0.96 | 99.9% |
| *04:07 | 50 | 1.2% | 0 | 0.0% | 0.00 | 98.8% |
| *04:08 | 43 | 1.0% | 22 | 0.5% | 0.65 | 99.4% |
| *04:11 | 1 | 0.0% | 0 | 0.0% | 0.00 | 100.0% |
| *04:34 | 1 | 0.0% | 0 | 0.0% | 0.00 | 100.0% |
| *07:01 | 551 | 12.9% | 556 | 13.0% | 0.99 | 99.9% |
| *08:01 | 95 | 2.2% | 102 | 2.4% | 0.96 | 99.8% |
| *08:02 | 5 | 0.1% | 0 | 0.0% | 0.00 | 99.9% |
| *08:03 | 4 | 0.1% | 0 | 0.0% | 0.00 | 99.9% |
| *08:04 | 7 | 0.2% | 13 | 0.3% | 0.73 | 99.9% |
| *08:10 | 2 | 0.1% | 0 | 0.0% | 0.00 | 100.0% |
| *08:11 | 1 | 0.0% | 0 | 0.0% | 0.00 | 100.0% |
| *09:01 | 49 | 1.2% | 46 | 1.1% | 0.97 | 99.9% |
| *10:01 | 58 | 1.4% | 59 | 1.4% | 0.97 | 99.9% |
| *11:01 | 175 | 4.1% | 259 | 6.1% | 0.77 | 97.7% |
| *11:02 | 10 | 0.2% | 8 | 0.2% | 0.78 | 99.9% |
| *11:03 | 27 | 0.6% | 0 | 0.0% | 0.00 | 99.4% |
| *11:04 | 103 | 2.4% | 49 | 1.1% | 0.59 | 98.5% |
| *11:12 | 3 | 0.1% | 0 | 0.0% | 0.00 | 99.9% |
| *11:13 | 1 | 0.0% | 0 | 0.0% | 0.00 | 100.0% |
| *11:39 | 1 | 0.0% | 0 | 0.0% | 0.00 | 100.0% |
| *12:01 | 47 | 1.1% | 48 | 1.1% | 0.95 | 99.9% |
| *13:01 | 210 | 4.9% | 219 | 5.1% | 0.97 | 99.7% |
| *13:02 | 202 | 4.7% | 201 | 4.7% | 0.99 | 99.9% |
| *13:03 | 48 | 1.1% | 46 | 1.1% | 0.98 | 100.0% |
| *13:04 | 1 | 0.0% | 0 | 0.0% | 0.00 | 100.0% |
| *13:05 | 4 | 0.1% | 0 | 0.0% | 0.00 | 99.9% |
| *14:01 | 87 | 2.0% | 63 | 1.5% | 0.82 | 99.3% |
| *14:04 | 3 | 0.1% | 29 | 0.7% | 0.32 | 99.4% |
| *15:01 | 565 | 13.2% | 563 | 13.2% | 0.99 | 99.9% |
| *15:02 | 35 | 0.8% | 36 | 0.8% | 0.99 | 100.0% |
| *15:03 | 1 | 0.0% | 0 | 0.0% | 0.00 | 100.0% |
| *16:01 | 57 | 1.3% | 66 | 1.5% | 0.93 | 99.8% |
| *16:02 | 9 | 0.2% | 0 | 0.0% | 0.00 | 99.8% |
| Overall |  |  |  |  |  | 93.1% |

**Supplementary Table 4.** Distribution of Mother-Child average Sequence Similarity Matching (SSM) Score by disease status

| **HLA locus** | **RA**  **(n=138)** | **SLE**  **(n=117)** | **Controls**  **(n=913)** |
| --- | --- | --- | --- |
| *A* |  |  |  |
| Mean ± SD | 21.0 ± 9.4 | 21.1 ± 9.0 | 20.8 ± 10.6 |
| Median | 22.1 | 22.4 | 23.2 |
| Range | 0-35.8 | 0-35.8 | 0-37.9 |
| *B* |  |  |  |
| Mean ± SD | 21.4 ± 7.7 | 21.0 ± 7.2 | 21.5 ± 8.3 |
| Median | 22.1 | 21.6 | 22.3 |
| Range | 0-37.2 | 0-35.4 | 0-37.2 |
| *C* |  |  |  |
| Mean ± SD | 14.2 ± 6.2 | 15.0 ± 5.9 | 13.9 ± 6.6 |
| Median | 16.0 | 17.0 | 15.5 |
| Range | 0-23.9 | 0-23.1 | 0-23.9 |
| *DPB1* |  |  |  |
| Mean ± SD | 4.6 ± 4.3 | 5.6 ± 3.8 | 5.1 ± 4.4 |
| Median | 2.9 | 5.5 | 2.9 |
| Range | 0-12.9 | 0-12.9 | 0-13.2 |
| *DQA1* |  |  |  |
| Mean ± SD | 3.5 ± 2.3 | 3.6 ± 2.4 | 3.5 ± 2.7 |
| Median | 3.4 | 3.9 | 3.3 |
| Range | 0-6.7 | 0-6.7 | 0-6.7 |
| *DQB1* |  |  |  |
| Mean ± SD | 12.2 ± 6.8 | 13.7 ± 6.5 | 13.3 ± 7.4 |
| Median | 12.8 | 14.2 | 13.9 |
| Range | 0-23.5 | 0-23.5 | 0-23.5 |
| *DRB1* |  |  |  |
| Mean ± SD | 13.0 ± 5.5 | 12.8 ± 5.0 | 13.3± 6.2 |
| Median | 13.0 | 13.0 | 13.0 |
| Range | 0-23.7 | 0-23.5 | 0-26.4 |
