# Supplementary figures and images for: Mother−child histocompatibility and risk of rheumatoid arthritis and systemic lupus erythematosus among mothers
Source: Genes Immun. 2019 Jan 12;21(1):27–36. doi: 10.1038/s41435-018-0055-7 (PMC7039805; doi:10.1038/s41435-018-0055-7)

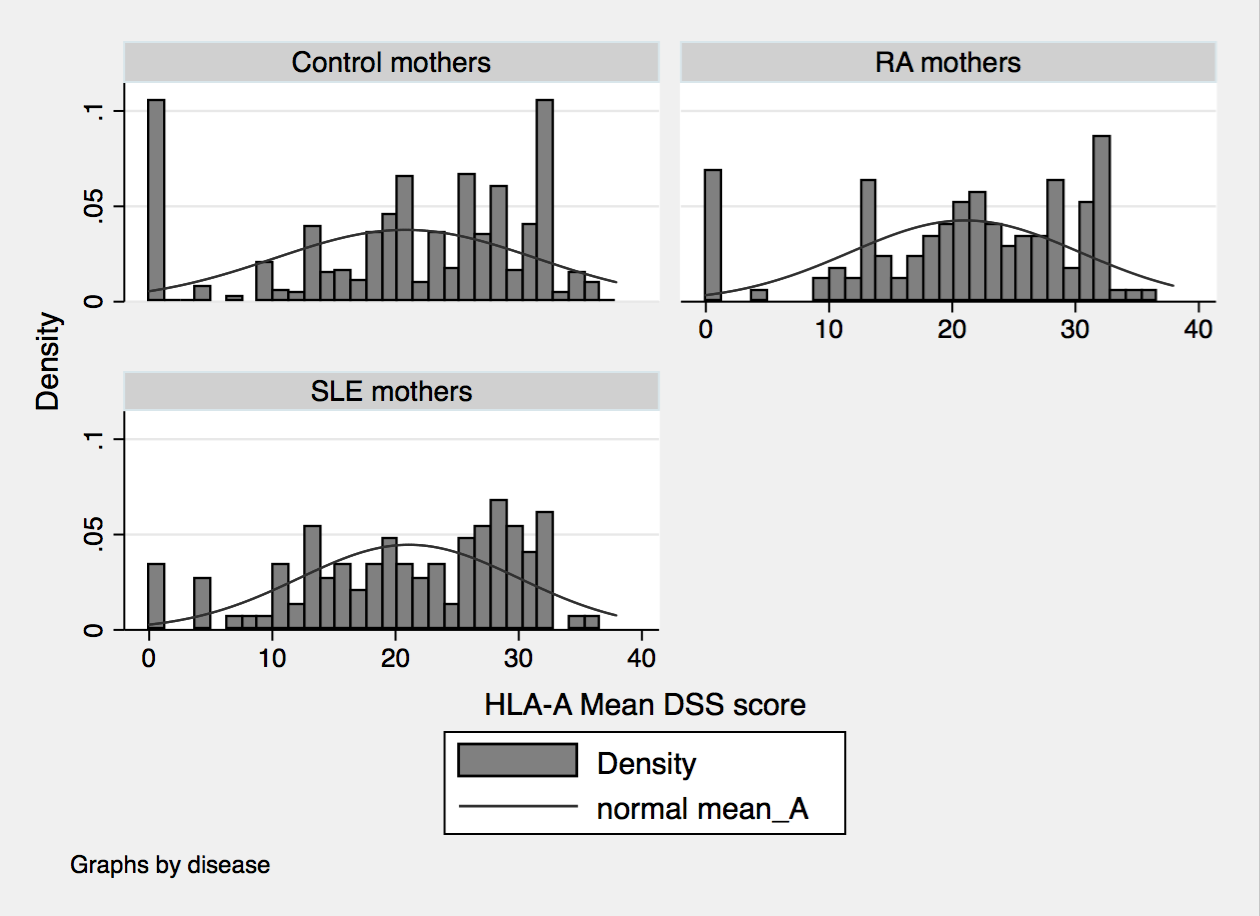

Supplement: Supplementary file 2 — Supplementary Figure 1. HLA-A Mother-Child average Sequence Similarity Matching (SSM) Score by disease status, (n=1,168) [file 41435_2018_55_MOESM2_ESM.tif]

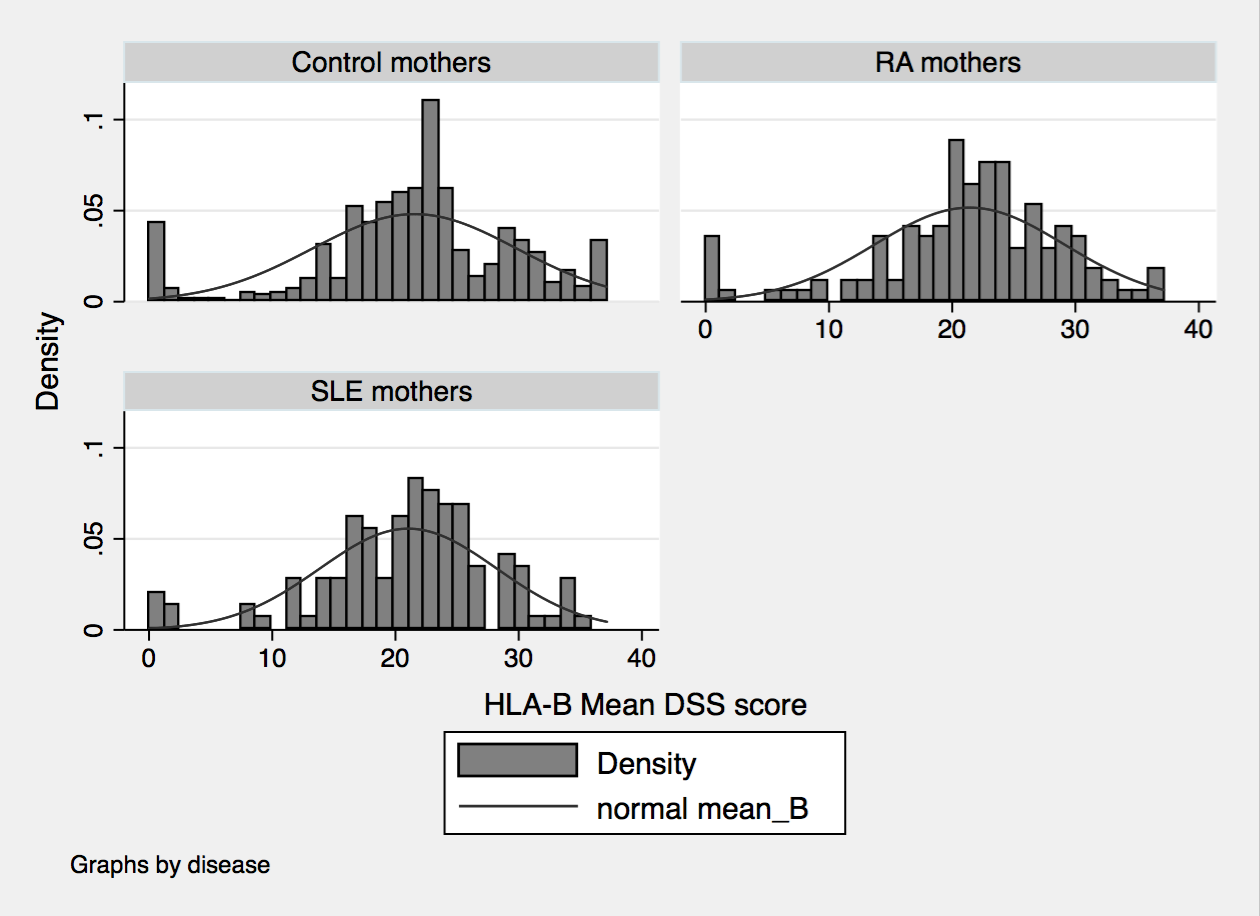

Supplement: Supplementary file 3 — Supplementary Figure 2. HLA-B Mother-Child average Sequence Similarity Matching (SSM) Score by disease status, (n=1,168) [file 41435_2018_55_MOESM3_ESM.tif]

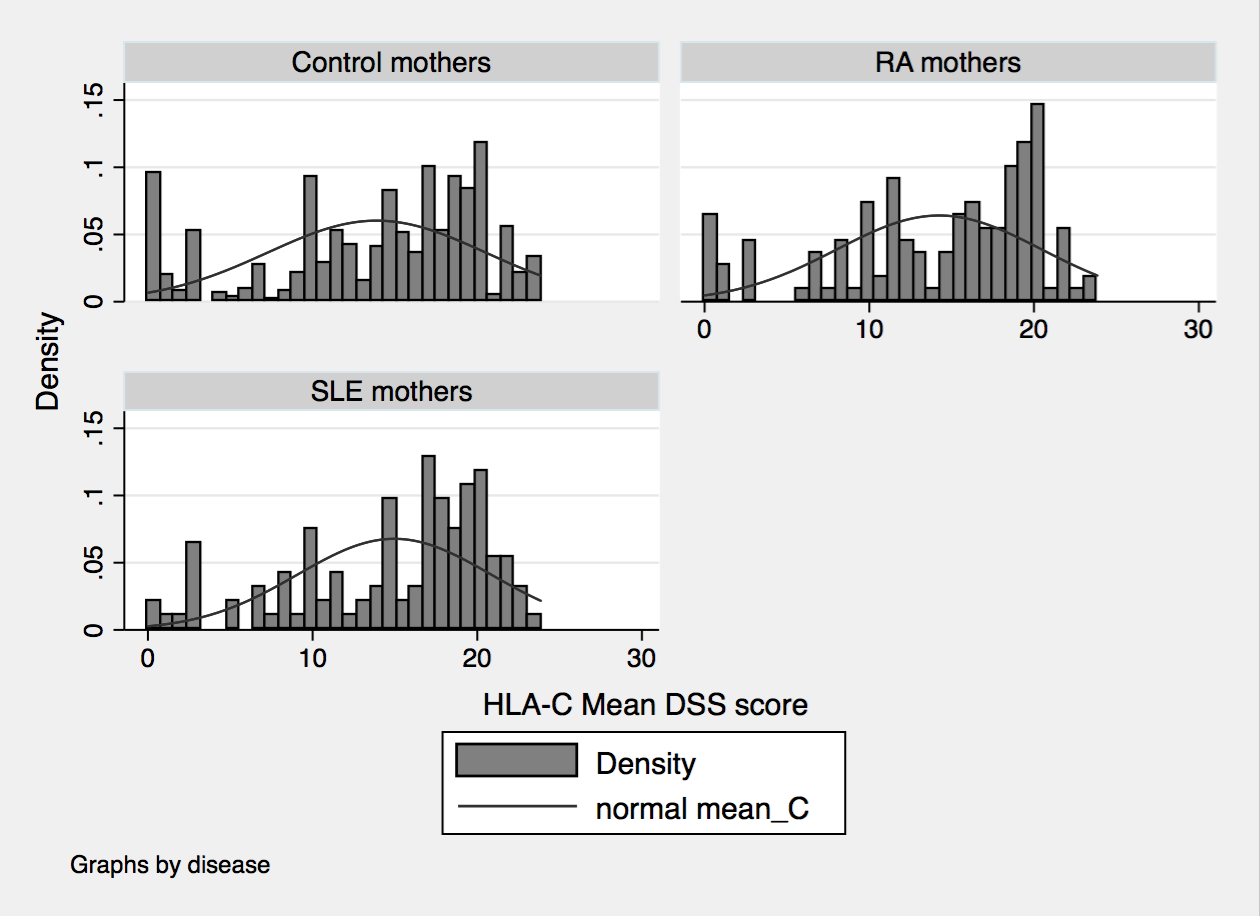

Supplement: Supplementary file 4 — Supplementary Figure 3. HLA-C Mother-Child average Sequence Similarity Matching (SSM) Score by disease status, (n=1,168) [file 41435_2018_55_MOESM4_ESM.tif]

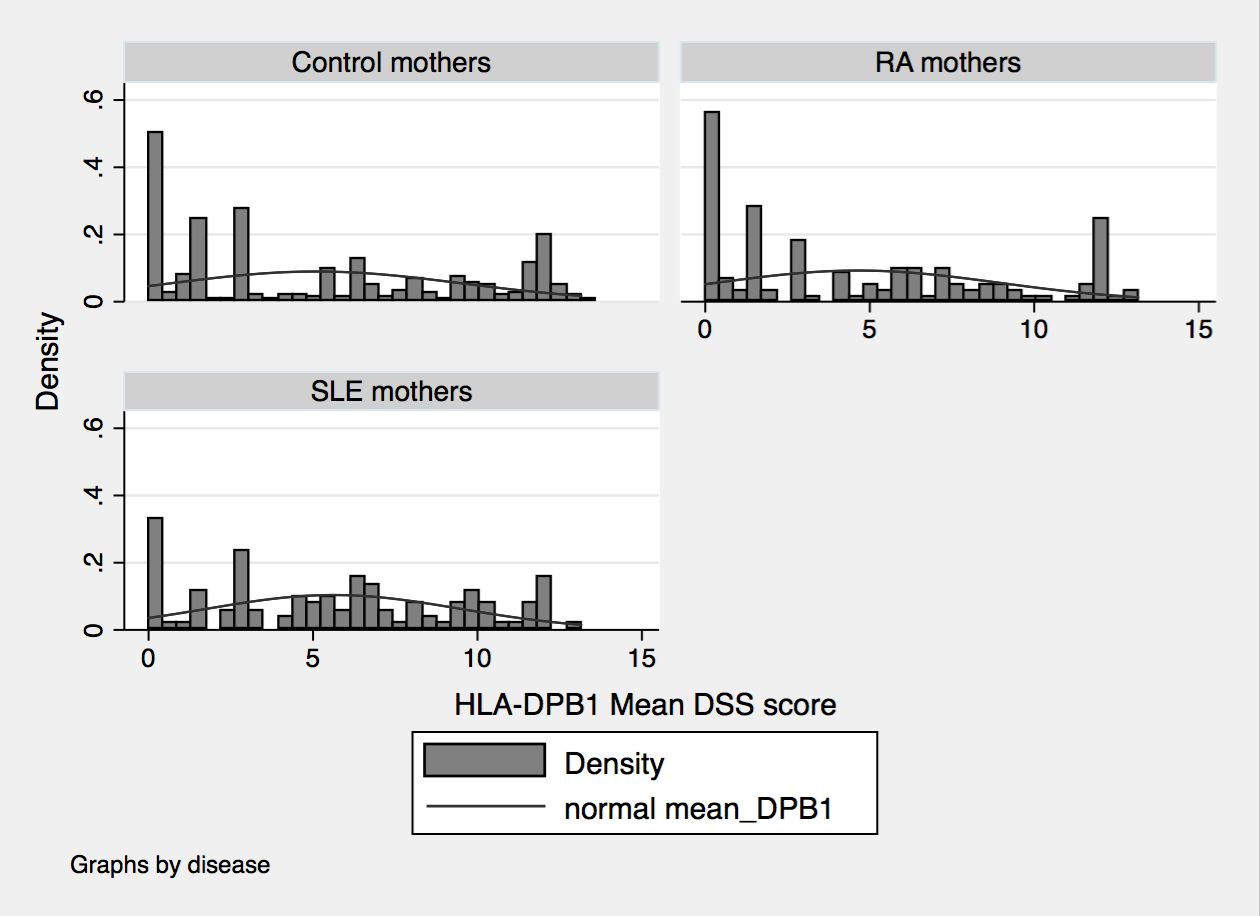

Supplement: Supplementary file 5 — Supplementary Figure 4. HLA-DPB1 Mother-Child average Sequence Similarity Matching (SSM) Score by disease status, (n=1,168) [file 41435_2018_55_MOESM5_ESM.tif]

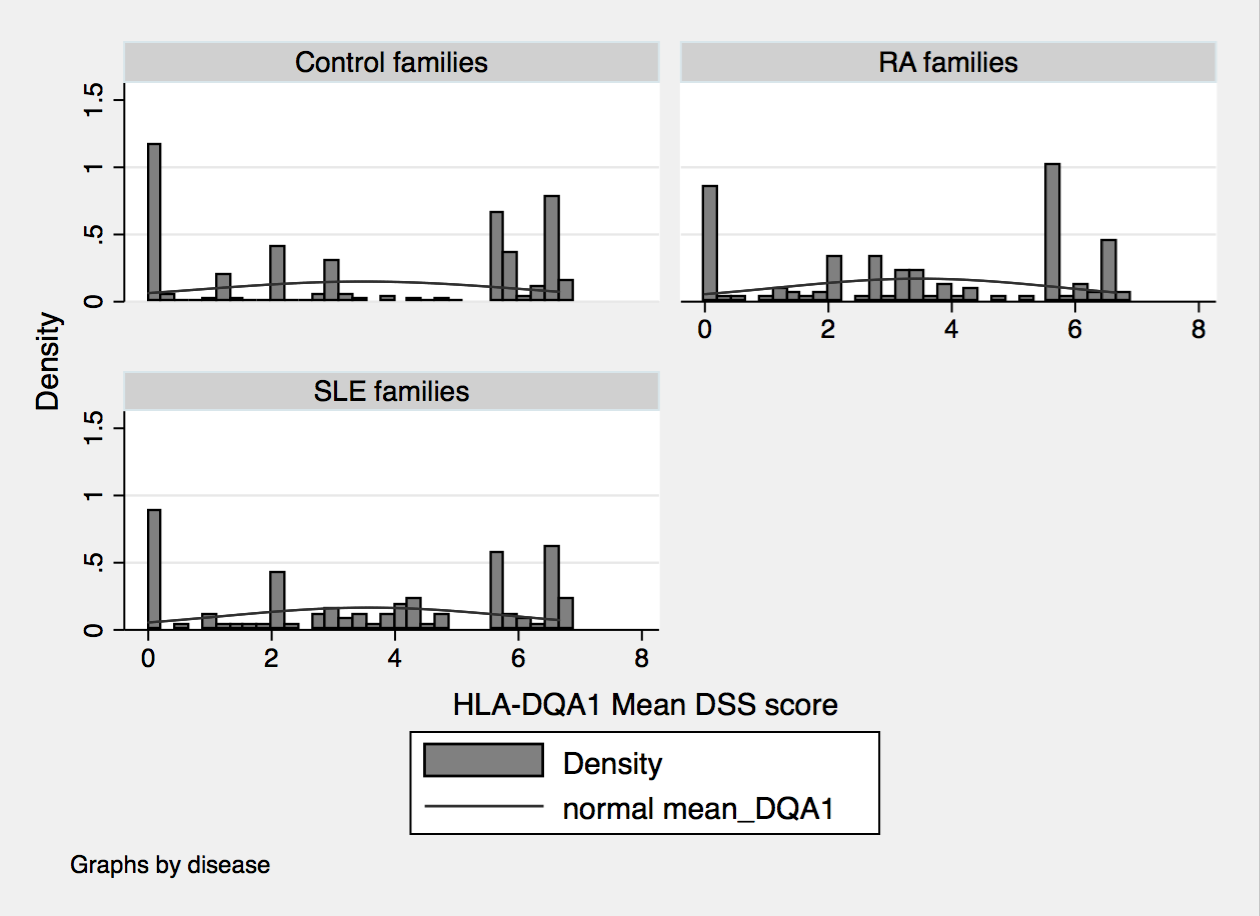

Supplement: Supplementary file 6 — Supplementary Figure 5. HLA-DQA1 Mother-Child average Sequence Similarity Matching (SSM) Score by disease status, (n=1,168) [file 41435_2018_55_MOESM6_ESM.tif]

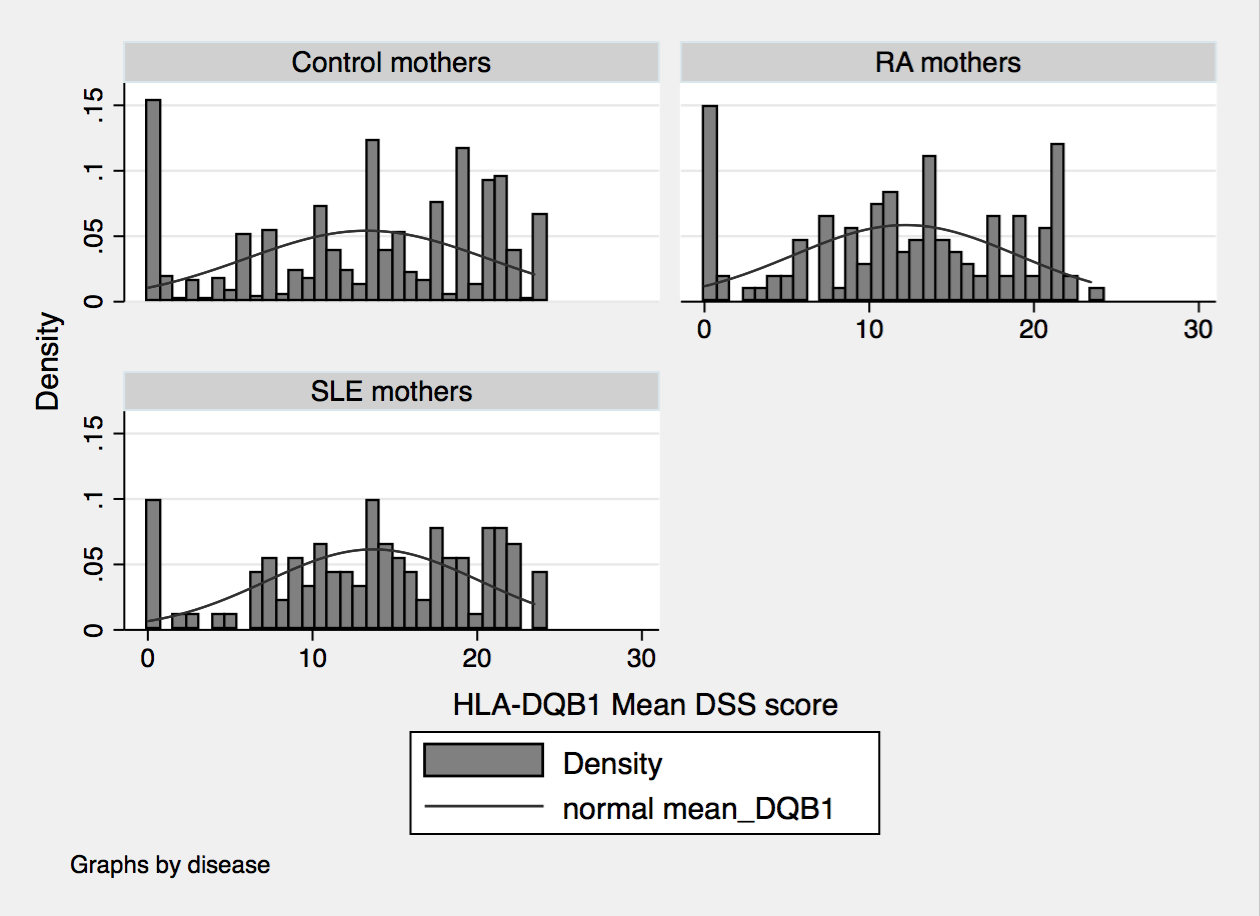

Supplement: Supplementary file 7 — Supplementary Figure 6. HLA-DQB1 Mother-Child average Sequence Similarity Matching (SSM) Score by disease status, (n=1,168) [file 41435_2018_55_MOESM7_ESM.tif]

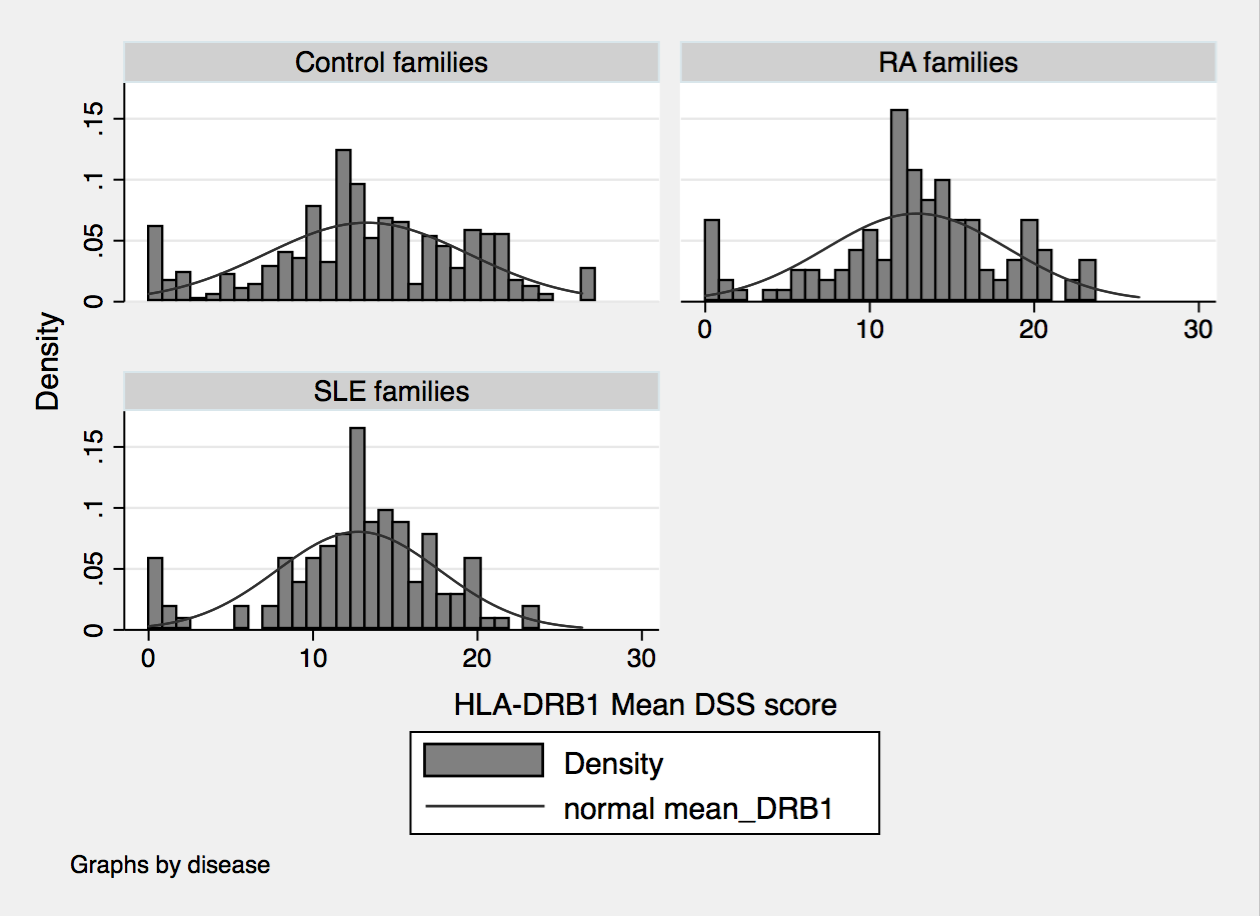

Supplement: Supplementary file 8 — Supplementary Figure 7. HLA-DRB1 Mother-Child average Sequence Similarity Matching (SSM) Score by disease status, (n=1,168) [file 41435_2018_55_MOESM8_ESM.tif]
